# Socio-economic variations of breast cancer treatment and discontinuation: a study from a public tertiary cancer hospital in Mumbai, India

**DOI:** 10.1186/s12905-023-02275-6

**Published:** 2023-03-20

**Authors:** Sanjay K. Mohanty, Tabassum Wadasadawala, Soumendu Sen, Pijush Kanti Khan

**Affiliations:** 1 Department of Population & Development, International Institute for Population Sciences, Mumbai, India; 2 Department of Radiation Oncology, Advanced Centre for Treatment, Research and Education in Cancer (ACTREC), Tata Memorial Centre, Homi Bhabha National Institute, Mumbai, India; 3 International Institute for Population Sciences, Govandi Station Road, Mumbai, India; 4 International Institute of Health Management Research, New Delhi, India

**Keywords:** Breast cancer, Treatment, Discontinuation, Survival, India

## Abstract

**Background:**

The study examined the socio-economic variation of breast cancer treatment and treatment discontinuation due to deaths and financial crisis.

**Methods:**

We used primary data of 500 patients with breast cancer sought treatment at India’s one of the largest cancer hospital in Mumbai, between June 2019 and March 2022. This study is registered on the Clinical Trial Registry of India (CTRI/2019/07/020142). Kaplan–Meier method and Cox-hazard regression model were used to calculate the probability of treatment discontinuation.

**Results:**

Of the 500 patients, three-fifths were under 50 years, with the median age being 46 years. More than half of the patients were from outside of the state and had travelled an average distance of 1,044 kms to get treatment. The majority of the patients were poor with an average household income of INR15,551. A total of 71 (14%) patients out of 500 had discontinued their treatment. About 5.2% of the patients died and 4.8% of them discontinued treatment due to financial crisis. Over one-fourth of all deaths were reported among stage IV patients (25%). Patients who did not have any health insurance, never attended school, cancer stage IV had a higher percentage of treatment discontinuation due to financial crisis. Hazard of discontinuation was lower for patients with secondary (HR:0.48; 95% CI: 0.27–0.84) and higher secondary education (HR: 0.42; 95% CI: 0.19–0.92), patients from rural area (HR: 0.79; 95% CI: 0.42–1.50), treated under general or non-chargeable category (HR: 0.60; 95% CI:0.22–1.60) while it was higher for the stage IV patients (HR: 3.61; 95% CI: 1.58–8.29).

**Conclusion:**

Integrating breast cancer screening in maternal and child health programme can reduce delay in diagnosis and premature mortality. Provisioning of free treatment for poor patients may reduce discontinuation of treatment.

## Introduction

Globally, breast cancer is now the leading cause of cancer accounting for 11.7% of 2.3 million new cancer cases [1]. In 2020, it accounts 7% (0.6 million) of 9.9 million all cancer deaths annually and the second leading cause of all oncological deaths [1]. In the recent decades, while there has been a decline in stomach, cervical and penile cancer, the incidence of breast, colorectal and prostate cancer has been rising [2]. By 2040, there would be three million new breast cancer cases diagnosed annually [3]. The prevalence, incidence and mortality from breast cancer varies enormously within and between the countries [1]. These variations may be attributed to a set of reproductive (early age at menarche, late menopause, childlessness, late childbearing, less breastfeeding), metabolic (weight, height, Body Mass I), lifestyle (diet, lack of physical activity, substance abuse etc.) and environmental and occupational factors (exposure to radiation, shift work involving disruption of circadian cycle etc.) [4–10]. Besides, advancement of medical technology, availability and accessibility to cancer screening and treatment have increased the diagnosis of this disease and its burden in low- and middle income countries (LMICs) [11].

Deaths due to breast cancer has long term consequences on growth and development of children and social well-being of families as mother, spouse, or daughter [12]. Breast cancer and its treatment deteriorate the physical, mental and functional health of the patient [13, 14]. The five-year survival of patients with breast cancer varies from 87% in United States, 84% in China, 83% in Sweden, 62% in Iran, 49% in Malaysia and 44% in Uganda [15–21]. Co-morbidity, depression among patients with breast cancer is very common including mild depression [22]. Overall, cancer patients have a lower quality of life (QoL).

Literature document strong socio-economic gradient of breast cancer screening, prevalence, survival and treatment discontinuation. The breast cancer screening and prevalence are lower among women belonging to low socio-economic status (SES) in LMICs [23, 24]. However, the risk of mortality from breast cancer is high among women belonging to low SES [17, 25]. The five-year survival rate of women belonging to poor SES was 78.6% compared to 83.8% for high SES [17]. Racial differences in survival of breast cancer are also large [26]. Treatment discontinuation is one of the major factors that affect the span of treatment and disease progression. It is often associated with symptoms exacerbation, relapse, comorbidity, death and higher economic burden at later stage [27]. Financial crisis due to high out-of-pocket expenditure for treatment of breast cancer is the major reason for treatment discontinuation [28].

Women in India are at a disadvantage, not only due to the high morbidity but also due to familial neglect of health care [29]. The disease burden due to breast cancer in India is higher than the world average. In 2020, India with 178,361 new cases (7.9% of global cases) and 90,408 deaths accounting for 13% of global mortality due to breast cancer only [1, 30]. Among women in India, breast cancer accounts 13.5% new cancer cases and 10.6% of cancer related mortality [30]. Many of the breast cancer mortality are premature and could have been saved with timely screening and medications [31, 32]. The age-standardized incidence rate of cancer among Indian females in 2018 was 90 per 100,000 females per year similar to males (89.8 per 100,000 males per year) [31]. Studies suggest that rising burden of breast cancer in the younger ages needs special focus in terms of reduction in treatment cost, quality management along with improved referral pathway and financial security against the disease.

The discontinuation of breast cancer is important as cancer patients often come from outside of the state to get treatment, it primarily affect women in working and reproductive age group, the mortality level is high and there has been rise in risk factors of breast cancer. The economic and social loss due to the disease is numerous to women, mother, children and family and also to the nation. To our knowledge no scientific study on treatment discontinuation of patients with breast cancer examined in Indian context. In this context, this paper examines the socio-economic variation in treatment discontinuation of the patients with breast cancer in a tertiary health care centre in India.

## Data & methods

### Study design

The study was a prospective non-interventional study.

### Study site

The study was conducted jointly by the Tata Memorial Centre (TMC), Mumbai, India and the International Institute for Population Sciences (IIPS), Mumbai, India. TMC is one of the country’s largest and the oldest public sector tertiary cancer hospital and registers more than 50,000 patients annually and IIPS is the leading demographic research and training center in the country. The study was designed by TMC and IIPS and the data collection was carried out at TMC.

### Sample design

TMC is one of the leading public sector tertiary care health center in India, located in Mumbai and dedicated to cancer treatment and research. Patients with cancer comes from all over the country and are often referred from other health centres on the country. The total number of patients registered for breast cancer treatment at TMC was 4,518 in 2019, 2,505 in 2020 and 3,588 in 2021. We have selected a total of 500 patients that account for about 8% of the breast cancer patients registered for treatment at TMC between June 2019 and March 2022. Our selection of 500 cases is guided by the fact that it provides enough power for disaggregated analyses of the economic condition of breast cancer patients and captures those treated under private and general category. According to WHO protocol, 200 samples is the minimum requirement for any health study [33]. Assuming 79% of catastrophic health spending by cancer households in India [34] and 95% confidence interval with 5% margin of error, a 255 sample size would require to estimate treatment-related cost. A similar procedure has been adopted in a Lancet study on catastrophic expenditure and treatment attrition among colorectal cancer patients in India [35]. Our sample size is about twice higher than the required sample. On average, 12 to 15 newly invasive breast cancer patients were registered every day in the hospital and 4 to 5 patients were selected randomly. The data was collected on working days only (excluding public holidays and weekends). There was disruption in data collection during March 2020-March 2021 due to COVID-19 restriction.

### Sample collection

Two separate questionnaires were developed and canvassed: a household questionnaire and an individual questionnaire. The household questionnaire covered demographic and socio-economic characteristics of a participant’s household at the time of registration at TMC. The individual questionnaire collected information on treatment history about current breast cancer diagnosis, treatment history at TMC, detailed record of the direct and in-direct health expenditure for each hospital visits during the entire course of treatment, comorbidities and self-rated health status of patients. Data collection was carried out by three medical social workers appointed in the project on daily basis. The data were collected and stored at TMC server and validated by principal investigators and researchers on weekly basis.

The data collection began from June 2019 and follow-up was continued till March 2022. Although during the planning of the study, it was decided to collect the baseline sample within one year period, but due to COVID-19 pandemic less patients were visited in the facility and hence the data collection timing shoots up to two years.

Data were collected for base line and selected questions during each visit of the treatment, at the end of treatment and six months after completion of treatment (termed as 1^st^ follow up). Often the data collection was contingent on the visit of patients for the services.

### Inclusion and exclusion criteria

The following inclusion criteria was set for data collection:

- Pathologically confirmed new invasive female breast cancer cases
- Intending to receive entire treatment at TMC
- Age > 18 years
- Patients willing to provide the information.

The exclusion criteria was as follows:

- Inability to follow up
- Recurrent or progressive breast cancer cases.

### Follow up

Each participant has been followed from their date of registration to their date of treatment conclusion or treatment discontinuation. The date of registration is termed as baseline whereas date of conclusion is termed as endline. An active follow up mechanism was set up to collect information from each of the participant or their accompanying person whenever they visit the facility.

### Censoring

We censored the data for 71 patients who had died or discontinued treatment for various reasons and not concluded in study time period. The discontinued patients were categorized in three major groups: discontinued due to death, discontinued due to financial crisis and other reasons (patients defaulted treatment or unable to contact patients).

### Survival time

Survival time is calculated at the time between the date of registration of the patients at TMC and conclusion date or date of last treatment just before the discontinuation.

### Other variables

A set of socio-demographic, economic and households’ variables were used in this study. These are age of the patient, level of education, marital status, financial dependent, health insurance coverage, patient’s category, family type, religion, major source of income, household income, household size, place of residence, state of residence, distance from native place. Age at diagnosis of cancer is the difference between date of cancer diagnosis and date of birth of the patient.

### Statistical analysis

Descriptive statistics, Kaplan–Meier survival estimation and cox proportional hazard model were used to examine the socio-economic profile and correlates of treatment discontinuation among the patients. Kaplan–Meier (KM) survival estimated the probability of discontinuation of breast cancer treatment. Each survival estimate used number of days under treatment at TMC as measure of time and whether patients discontinued (yes = 1 or no = 0) as the final event (failure). The KM estimate of survival time S(t) is given by:

$$\mathrm{S}(\mathrm{t})={\prod }^{\mathrm{k}}\mathrm{i}=1\frac{(ni-di)}{ni}$$

where n_i_ is the number of patients observed at time t_i_, and d_i_ is the number of patients discontinued at time t_i_. Cox-proportional hazard model was used to examine the socio-economic correlates of patient’s treatment discontinuation at TMC. The model estimates the risk of a patient to discontinue the treatment at time t, given that, the patient continued treatment up to time. The final event of interest for each of the patient was status of their treatment (discontinued = 1 or continued = 0) within the given span of time. The Cox proportional hazard model is specified by:

$$\frac{hi (ti;xi)}{h0 (ti)}=\mathrm{ exp }({\upbeta }^{\mathrm{i}}{\mathrm{x}}_{\mathrm{i}})$$

where h_i_ (t_i_; x_i_) is the hazard function of discontinued patients at time t_i_ to the patients who continued the treatment given the specified set of independent variables denoted as x_i_. and β_i_ is the parameters to be estimated.

### Ethical consideration

The study obtained approval from the institutional ethics committee of the TMC and was registered on the Clinical Trial Registry of India with CTRI No CTRI/2019/07/020142 on 10/07/2019.

## Results

Table 1 shows the sample characteristics of 500 patients with breast cancer undergoing treatment at TMC. About 5.6% were under 30 years, 57.4% were between 31–50 years, and 37% were 50 years and older. The youngest age at diagnosis was as early as 21 years. The mean years of schooling were 7 years and over four-fifths of the patients were married and financially dependent. Only 9% of the patients were covered by any health insurance scheme. Majority (85%) of the patients were registered under the general category to get treatment at TMC. The majority of the patients belonged to the Hindu religion (78%), and 52% were from the unreserved social class. More than half of the patients were from outside of the state of Maharashtra. On average, a patient travelled 1,044 kms to get breast cancer treatment at TMC. Half of the patients were inhabitants of rural areas (53%) and 16% resided in urban slums. Of the 500 patients registered for treatment, 429 patients had concluded their treatment at TMC. The socio-economic variation of the concluded patients almost remains the same as of the baseline.

**Table 1:** Sample profile of the patients with breast cancer seeking treatment at TMC, 2019–21

	Baseline		Endline	
SES variables	*N* = 500	%	*N* = 429	%
**Patients’s characteristics**				
**Age**				
Below 30	28	5.6	24	5.6
31 to 40	124	24.8	111	25.9
41 to 50	163	32.6	145	33.8
51 to 60	134	26.8	108	25.2
60 above	51	10.2	41	9.6
**Years of schooling**				
Never attended	133	26.6	99	23.08
Primary	43	8.6	36	8.39
Secondary	186	37.2	167	38.93
Higher secondary	56	11.2	50	11.66
Above higher secondary	82	16.4	77	17.95
**Marital Status**				
Currently married	422	84.4	366	85.31
Other	78	15.6	63	14.69
**Financial dependent**				
Yes	425	85	365	85.08
No	75	15	64	14.92
**Health insurance**				
Yes	46	9.2	38	8.86
No	454	90.8	391	91.14
**Patients’ category at baseline**^**a**^				
Non-chargeable	6	1.2	23	5.36
General	429	85.8	346	80.65
Private	65	13	60	13.99
**Household characteristics**				
**Type of current residence**				
Own house	143	28.6	121	28.2
Relative/friend’s house	115	23	97	22.6
Rented room	157	31.4	140	32.6
Hotel	28	5.6	24	5.6
Ashram and others	57	11.4	47	11
**Family type**				
Nuclear family	287	57.4	249	58.04
Non-nuclear family	213	42.6	180	41.96
**Religion**				
Hindu	394	78.8	332	77.39
Muslim	86	17.2	80	18.65
Other	20	4	17	3.96
**Major source of household income**				
Labour	129	25.8	103	24.01
Self-employed	79	15.8	66	15.38
Service and pension	228	45.6	206	48.02
**Household size**				
1 to 4	248	49.6	217	50.58
5 to 6	179	35.8	155	36.13
7 and more	73	14.6	57	13.29
**Mean household size**		**4.85 (1.9)**		**4.8 (1.9)**
**Residence**				
Urban	232	46.4	196	45.69
Rural	268	53.6	233	54.31
**State**				
Maharashtra	227	45.4	237	55.24
Outside of Maharashtra	273	54.6	192	44.76
**Distance from native place to Mumbai**				
upto 500 kms	217	43.4	185	43.12
501 to 2000 kms	186	37.2	156	36.36
over 2000 kms	97	19.4	88	20.51
**Total**	**500**	**100**	**429**	**100**

^a^At TMC, the patients are classified as a) general b) private and c) non-chargeable. During the registration, a patient registers either as a general or as a private patient depending on their ability to pay for treatment. The cost of treatment for private patient category is much higher while the waiting time for availing treatment is much lower than their other counterpart

**Fig. 1:**
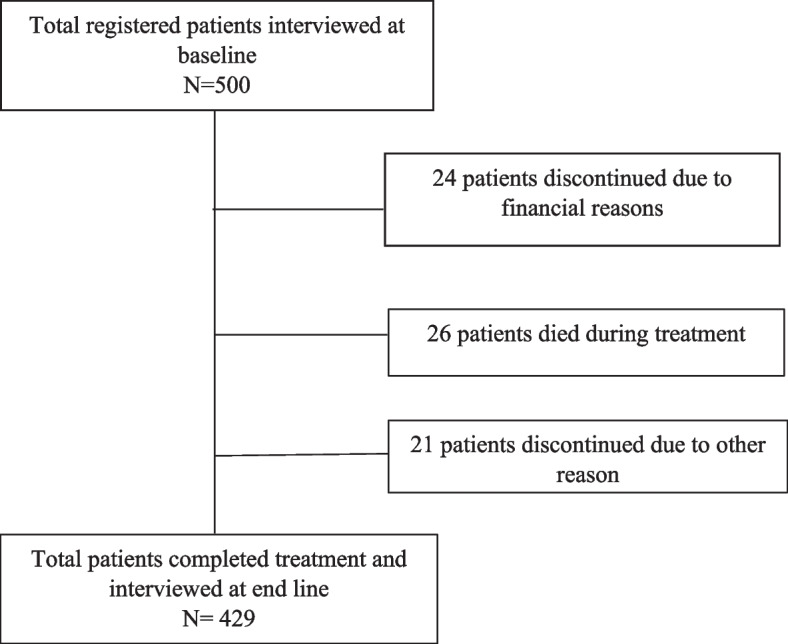
Flow chart of breast cancer treatment discontinuation at TMC, 2019–22

Figure 1 presents the flow chart of the patients discontinued and the reason of discontinuation. About 71 patients discontinued their treatment. Among them 24 patients discontinued due to financial reason, 26 due to deaths and 21 for other reason. A total 429 patients had completed the treatment.

Table 2 presents the socio-economic variation of the patients who discontinued treatment at TMC. Overall, 14.2% of the patients were discontinued the treatment and deaths (37%) being the major cause of treatment discontinuation followed by financial crisis (34%). A higher percentage of the patients discontinued treatment were over 46 years (15.9%), never attended schooling (25.6%), not currently married (19.2%), treated under general or non-chargeable category (14.9%), diagnosed at stage IV of cancer (46.4%), had atleast one comorbidity (19.7%) and labour as major source of household income (20.2%). Among those who discontinued treatment, death accounts the largest share (37%) followed by financial reason (34%). The highest discontinuation due to financial reason were reported by the households with self-employment (54%), followed by service and pension (32%). The higher discontinution due to death were reported by households with agricultural income (50%) followed by self-employed (39%). Discontinution due to financial reason was higher in urban areas (36%) while, discontinued treatment due to death was higher in rural areas (54%).

**Table 2:** Percentage of the patients with breast cancer discontinued treatment at TMC

				Discontinued by reason among discontinued patients		
**Patients’s characteristics**	Number of patients discontinued	% Discontinued	*p*-value (log-rank test)	Financial reason	Death	Others
**Age**						
45 and below	28	12.2	0.091	35.7	39.3	25.0
46 and above	43	15.9		32.6	34.9	32.6
**Years of schooling**						
Never attended	34	25.6	0.007	38.2	32.4	29.4
Up to secondary	26	11.4		38.5	42.3	19.2
Higher Secondary and above	11	8.0		9.1	36.4	54.5
**Marital Status**						
Currently married	56	13.3	0.352	33.9	35.7	30.4
Other	15	19.2		33.3	40.0	26.7
**Health insurance**						
Yes	7	15.6	0.866	0.0	57.1	42.9
No	64	14.1		37.5	34.4	28.1
**Patients category:_Baseline**						
General/Non chargeable	65	14.9	0.482	35.4	38.5	26.2
Private	6	9.2		16.7	16.7	66.7
**Stage**						
I-II	13	7.7	0.009	46.2	23.1	30.8
III	45	14.8		35.6	35.6	28.9
IV	13	46.4		15.4	53.8	30.8
**Self-reported financial condition**						
Good	3	6.8	0.084	0.0	66.7	33.3
Moderate	24	13.1		41.7	20.8	37.5
Poor	44	16.1		31.8	43.2	25.0
**Diagnosed with cancer**						
Within 1 month	28	13.1	0.478	35.7	32.1	32.1
More than 1 month	43	15.0		32.6	39.5	27.9
**Comorbidity status**						
No comorbidity	47	12.4	0.047	31.9	38.3	29.8
At least 1 morbidity	24	19.7		37.5	33.3	29.2
**Household’s characteristics**						
**Family type**						
Nuclear family	38	13.2	0.366	23.7	36.8	39.5
Non-nuclear family	33	15.5		45.5	36.4	18.2
**Religion**						
Hindu	62	15.7	0.130	33.9	37.1	29.0
Muslim/ others	9	8.5		33.3	33.3	33.3
**Social Group**						
General	33	12.7	0.627	30.3	36.4	33.3
OBC	24	14.2		41.7	33.3	25.0
SC/ST/Other	14	19.4		28.6	42.9	28.6
**Major source of household income**						
Agriculture	10	15.6	0.255	30.0	50.0	20.0
Labour	26	20.2		26.9	34.6	38.5
Self-employed	13	16.5		53.8	38.5	7.7
Service and pension	22	9.6		31.8	31.8	36.4
**Household size**						
1 to 4	31	12.5	0.383	16.1	45.2	38.7
5 to 6	24	13.4		37.5	33.3	29.2
7 and more	16	21.9		62.5	25.0	12.5
**Residence**						
Urban	36	15.5	0.034	36.1	19.4	44.4
Rural	35	13.1		31.4	54.3	14.3
**State**						
Maharashtra	36	13.2	0.009	30.6	50.0	19.4
Outside of Maharashtra	35	15.4		37.1	22.9	40.0
**Distance from native place to Mumbai**						
Upto 500 km	30	14.0	0.114	43.3	13.3	43.3
501 and above	41	14.4		26.8	53.7	19.5
**Total**	**71**	**14.2**		**33.8**	**36.6**	**29.6**

**Fig. 2:**
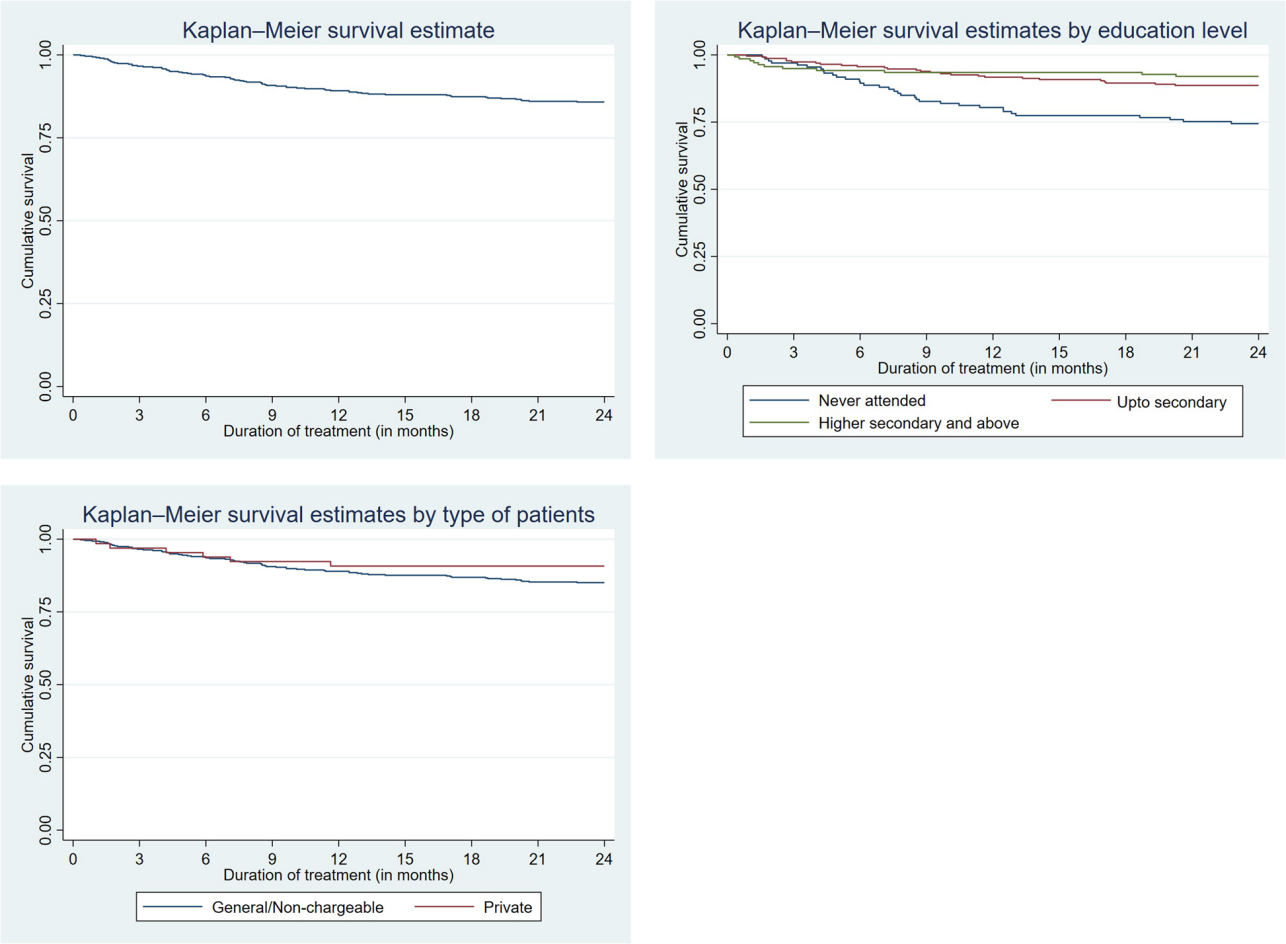
Kaplan–Meier survival curve for (**a**) discontinuation of treatment at TMC (**b**) discontinuation by education (**c**) by type of patient

Figure 2 shows the Kaplan–Meier survival curve for the discontinued patients at TMC with overall survival rate is 85.8%. Patients who sought treatment under general or non-chargeable category had higher probability of discontinuing treatment than the private patients.

Table 3 presents the hazard rates of discontinued patients by socio-economic variables. The estimated hazard shows that compared to the patients aged 45 years and below, patients aged 46 years and above were more likely (HR: 1.14, 95% CI: 0.66–1.97) to discontinue treatment. Similarly, patients who are not currently married, had advanced stage of cancer, had at least one comorbidity, belonged to household size 7 and more and from Maharashtra were more likely to discontinue treatment at TMC. On the other hand, patients with education level up to secondary (HR: 0.48; 95% CI: 0.27–0.84) and higher secondary or above (HR: 0.42; 95% CI: 0.19–0.92) were significantly less likely to discontinue the treatment.

**Table 3:** Hazard ratio and 95% confidence interval of patients discontinued treatment at TMC

Socio economic variables	Hazard Ratio	95% CI
**Age**		
45 and below ®	1	
46 and above	1.14	0.66–1.97
**Years of schooling**		
Never attended	1	
Up to secondary	0.48**	0.27–0.84
Higher Secondary and above	0.42**	0.19–0.92
**Marital Status**		
Others ®	1	
Currently married	1.19	0.62–2.29
**Health insurance**		
Yes ®	1	
No	0.92	0.35–2.39
**Patients category baseline**		
Private ®	1	
General/Non	0.60	0.22–1.60
**Stage**		
I-II	1	
III	1.45	0.76–2.77
IV	3.61**	1.58–8.29
**Diagnosed with cancer**		
Within 1 month	1	
More than 1 month	1.19	0.72–1.96
**Comorbidity status**		
No comorbidity	1	
At least 1 morbidity	1.50	0.85–2.62
**Religion**		
Hindu	1	
Muslim/others	0.46*	0.22–0.97
**Social Group**		
General	1	
OBC	0.60	0.34–1.07
SC/ST/Other	0.94	0.47–1.88
**Income range**		
Upto 10 k	1	
10 k-25 k	0.77	0.41–1.43
25 k-50 k	0.50	0.18–1.38
50 k +	0.25	0.05–1.18
**Household size**		
1 to 4	1	
5 to 6	1.52	0.86–2.69
7 and more	2.05*	1.00–4.21
**Residence**		
Urban	1	
Rural	0.79	0.42–1.50

^*^ < 0.05, ** < 0.01

## Discussion

The rising prevalence of breast cancer is a major public health challenge in India. There is dearth of comprehensive studies on socio-economic and health condition of the patients with breast cancer seeking active oncological treatment and reasons for discontinuation. We document the socio-demographic and economic profile of treatment discontinuation of breast cancer patients undergoing active cancer treatment. The followings are the salient findings of the study.

About three-fifths of the patients with breast cancer were under 50 years and the median age was 47 years. This is suggestive that majority of the patients are in child bearing age and a significant proportion are in prime child bearing ages (30% under 40 years). Cancer to young mothers has adverse implications on child breast feeding, rearing and caring of children. Besides, over 90% are under 60 years of age suggesting that it may adversely reduce the work potential. These findings are consistent with literature suggesting that the average age is low compared to developed countries; more than 50 years in USA and Europe [36, 37].

We found about 14% discontinued treatment and the discontinuation was significantly higher among poorer women, less educated and older women. The discontinuation is primarily due to three reasons; death, financial crisis and defaulted. During two-year period, 5% discontinued due to financial crisis and 5% died. The survival rate was little higher as it was for two-year reference. Studies suggest five-year survival rate ranging from 20 to 80% in India [21]. The discontinuation of services due to death was higher among poor, less educated and those in higher stage III/IV of breast cancer. Studies suggest that half of the incidence and mortality of breast cancer is under 50 years in countries with a lower Human Development Index (HDI) [38–40]. Discontinuation of services due to financial reason were also higher among poor and less educated. Our findings suggests that the economic condition of majority of the patients with breast cancer was poor. Over 86% patients were treated as general or non-chargeable cases. Moreover, during the COVID-19 pandemic the financial situation of most of the patients worsened significantly [41].

Our study also showed that more than half of the patients travelled from outside of the state. Distance is an important factor for cancer patients to continue treatment as they have to make frequent visits to the hospital during the course of treatment [42]. Studies also suggest that distance is negatively associated with the stage of diagnosis, appropriate treatment and outcomes, and quality of life [41, 43–45]. Distance majorly contributes to the increased cost of travel and arranging accommodation close to the hospital. The effect is more prominent for patients coming from rural areas. Cancer treatment facilities in India are limited in number and mostly metro-city centric. The socially and economically disadvantaged population from rural areas faces numerous challenges in access to cancer treatment. Patients from remote and rural areas travel long distances for treatment, which has a significant effect on their economic and health status.

Health insurance plays a significant role in reducing financial burden. We found lower coverage of insurance among the patients compared to entire population [46]. In 2018, the Government of India launched a comprehensive cashless health insurance scheme, *Ayushman Bharat*, for the bottom 40% of the population, providing ₹500,000 per family per year for health care expenditure. This scheme has the potential to deliver quality health care for cancer by linking reimbursement directly to the evidence-based management guidelines recommended by India’s National Cancer Grid, which is important for a disease where affordability of treatment is a big issue [47, 48]. According to a recent study, there are 1575 hospitals in India where cancer treatment costs can be reimbursed through this scheme; however, only 438 hospitals, including TMC, have multimodality treatment facilities [47].

We have some limitations of this study. First, this study is limited to patients treated at a single tertiary care hospital and may not be generalised. Though TMC cater services to all economic group, we believe that majority patients treated at TMC are poor. Second, we have not followed up patients for longer duration. The breast cancer associated mortality would have been higher in five-year period. Third, some of the patients who left discontinued treatment could not be contacted. Fourth, the study period also coincides with the COVID-19 pandemic which might affect the economic condition of the households of the study participants.

## Conclusions

Our study called for free screening of breast cancer for socially and economically disadvantageous women in public health centres and strengthening referral mechanism for early treatment of the patients with breast cancer. The health infrastructure for female cancer screening and treatment is inadequate and even non-existent in many states of India. Public investment on cancer treatment in terms of providing financial safety net will benefit women and reduce the burden of the disease. Health insurance should not only reduce the out-of-pocket burden for the treatment but should encourage the patients to continue their planned treatment in respective facilities.

## Data Availability

The datasets used and/or analysed during the current study is not publicly available and cannot be shared. However, one may contact Dr. Tabassum Wadasadawala, ACTREC Navi Mumbai, email: twadasadawala@actrec.gov.in for the study data and upon reasonable request the data may be shared. It may be noted that Dr. Wadasadawala has every right to make decision on data sharing.

## Abbreviations

LMIC: Low and Middle Income Country
QoL: Quality of Life
SES: Socio-economic Status
IIPS: International Institute for Population Sciences
TMC: Tata Memorial Center
HDI: Human Development Index
